# Supplementary material for: Nurturing families: One year pilot outcomes for a modified Parent Child Assistance Program in Australia
Source: PLOS Glob Public Health. 2022 Aug 10;2(8):e0000580. doi: 10.1371/journal.pgph.0000580 (PMC10022323; doi:10.1371/journal.pgph.0000580)
Supplement: Attachment — Submitted filename: Response to Reviewers PCAP Paper 1 v3.docx [file pgph.0000580.s001.docx]

Response to Reviewers

Journal Requirements:

1. Please update your Competing Interests statement. If you have no competing interests to declare, please state: “The authors have declared that no competing interests exist.”

Reviewers' comments:

Reviewer's Responses to Questions

The authors have declared that no competing interests exist. This work was funded by the Australia National Health and Medical Research Council to the FASD Research Australia Centre of Research Excellence (Grant number 1110341).

**Comments to the Author**

1. Does this manuscript meet PLOS Global Public Health’s [publication criteria](https://journals.plos.org/globalpublichealth/s/criteria-for-publication)? Is the manuscript technically sound, and do the data support the conclusions? The manuscript must describe methodologically and ethically rigorous research with conclusions that are appropriately drawn based on the data presented.

Reviewer #1: Yes

Reviewer #2: Yes

2. Has the statistical analysis been performed appropriately and rigorously?

Reviewer #1: Yes

Reviewer #2: I don't know

3. Have the authors made all data underlying the findings in their manuscript fully available (please refer to the Data Availability Statement at the start of the manuscript PDF file)?

The [PLOS Data policy](https://journals.plos.org/globalpublichealth/s/data-availability) requires authors to make all data underlying the findings described in their manuscript fully available without restriction, with rare exception. The data should be provided as part of the manuscript or its supporting information, or deposited to a public repository. For example, in addition to summary statistics, the data points behind means, medians and variance measures should be available. If there are restrictions on publicly sharing data—e.g. participant privacy or use of data from a third party—those must be specified.

Reviewer #1: Yes

Reviewer #2: No

In our cover letter we outlined the reasons why data could not be made publicly available. Due to the small sample size and inclusion of questions regarding illegal activities such as drug use, to best preserve the anonymity of clients and protect their interests we assured them that their data would not be shared.

4. Is the manuscript presented in an intelligible fashion and written in standard English?

PLOS Global Public Health does not copyedit accepted manuscripts, so the language in submitted articles must be clear, correct, and unambiguous. Any typographical or grammatical errors should be corrected at revision, so please note any specific errors here.

Reviewer #1: Yes

Reviewer #2: Yes

5. Review Comments to the Author

Please use the space provided to explain your answers to the questions above. You may also include additional comments for the author, including concerns about dual publication, research ethics, or publication ethics. (Please upload your review as an attachment if it exceeds 20,000 characters)

Reviewer #1: To the Authors

Thank you for the opportunity to review your paper. The paper offers valuable insights into the value of the PCAP program in an Australian context. The paper is well-written and neatly organised. I have listed some recommendations to strengthen your paper.

Thanks for your kind words of support for our paper and for your suggestions about improvements which we have incorporated into the paper.

Page 12: Please clarify if the implementers received or did not receive the free training plan as you indicate that they did not receive the free plan but subsequently explain that they did. Later in the paper you indicate that they did not participate in formal training but had access to the free manuals. Perhaps I am misunderstanding, but please try to clarify.

The case-managers did read the freely available manuals. However, they did not undertake the in-person training available in the US which would typically be a requirement of becoming a case-manager. At no point in the manuscript can we find any mention of them attending training.

We have made this clearer by amending the text at line 264 (page 13) onwards to read: “The case-managers did not receive the free in-person official PCAP training program available in the US because of budgetary restraints. They were required to: read and understand the freely available online PCAP manuals, discuss the implementation of the program with the program manager, and watch two unofficial training videos available on YouTube.” We also made it clear on line 41-42 that they did not undertake in person training and relied on the training manuals. Also, at line 507 that case managers wished to attend the in-person training.

P24- 4.11: It would be useful to draw up a table with some qualitative data that describe the case managers perceptions of the programme.

On the recommendations of Reviewer 2, we have decided to remove the case-manager aspect of this paper and expand on both that and provide more information about service delivery aspects in another paper. This includes removal of the short description of the methods at line 232 (p.11) and results at line 503 (p. 24). We have included a mention of these further results being made available in another paper in the discussion at line 616 (page 29), “Further examination of these contextual issues, case-manager perceptions of the program and the full suite of implementation details will be undertaken after the first cohort of clients finish the full three year program by applying the Consolidated Framework for Implementation Research (CFIR).”.

General questions
- Did you observe any age related differences between adult youth and middle adults

Due to our small sample size we didn’t contemplate looking at subsets within the data. As an attempt to preserve some anonymity in client data in case it was subpoenaed for a court case five-year age ranges were used. As two clients were aged 25-29, three from 30-34 and three from 35-39 (line 155) we don’t feel there is enough people in each group to usefully make comparisons.

As this is an important issue we did add this to the discussion from line 693 (page 32), “It is currently not known if the PCAP program will differ in effectiveness for those who are younger or have had difficulties with AOD over shorter periods and those who are older or have longer AOD histories. This is an important question to consider as in Australia women with moderate and high alcohol use during pregnancy were more likely to have first become intoxicated before the legal age of 18 and high sustained drinking more common in women over 35 (Muggli, 2022).”

- Could you comment on any contextual issues you might have observed or be aware of that could have influenced experiences of the program?

We have included a note about the experiences of domestic violence that may have impacted on program experience as it could limit the time spent with case-managers, and topics discussed during their meetings. We have added the following text to the discussion at line 632, page 28. “There were contextual issues that may have affected experiences of the program for some clients including experiences of domestic violence and availability of services in the different areas in which clients lived. For those clients currently needing domestic violence services (5 out of 8), depending on the type and behaviour by the partner, it may have limited the interactions with the case-manager both in terms of time spent together and the topics of discussion when meeting.“.

To support this we added the number of women accessing domestic violence services at line 412, page 19, “At intake two women were accessing domestic violence services and at follow-up four were currently accessing services with one in extreme need.”.

We have also added a note about access to services directly after at line 612, page 29, “Also, clients at times reported their interactions with various services available as poor. If there were no practical alternatives available locally they may not have been able to access services with good fit which may have led to reduced therapeutic benefit. At times, good services clients were attending lost funding and were closed.”

Further issues will be explored in a later paper.

- How do the findings compare with other contexts?

We have added the following paragraph to the results at line 548, page 26 “The characteristics of women who took part in our study and their outcomes in terms of reducing risk of an AOD exposed pregnancy were similar to other studies where the PCAP has been trialled in the US (Grant, 2005) and Canada (Umlah, 2003). To our knowledge this was the first time changes in areas in which clients expressed concern and need for further treatment was reported for PCAP. Similar programs are rare or not often evaluated. A recent systematic review of programs for prevention of prenatal alcohol exposure and FASD identified PCAP as the only long-term support program found effective in evaluations, with four other programs showing some short-term reductions that were not sustained (Erng, 2020). Short-term brief interventions were the most popular approach and while they showed some promise for reducing alcohol exposed pregnancies, only four recruited from women at higher risk of PAE and only one had a follow-up period of 12 months, with the other 14 having follow-ups of 6 months or less, making long-term benefits uncertain (Erng, 2020). No other studies found were conducted in Australia (Erng, 2020). As prevention of alcohol use during pregnancy has unique opportunities and challenges, it is not appropriate to make comparisons with approaches designed to reduce general alcohol use.

- How do the findings compare to other/similar interventions or programs aimed to collectively or individually address the issues targeted in the paper? Are there other programs or elements of other programs that could be incorporated in future work that could potentially strengthen the program or making more contextually relevant?

This program is unique in terms of the intensity and length of time support is provided to clients. In the previous point we have summarised findings from a systematic review of prenatal alcohol exposure prevention programs.

Additionally, we have added the following paragraph to the discussion at line 684, page 31 “Given many PCAP clients experience mental health issues researchers in the US have hypothesised that the program may be strengthened by providing case-managers with further training in this area. Therefore, they have conducted a promising trial addition of an adapted version of the culturally relevant MomCare mental health program [52] which appeared to reduced depression in clients and applied for further funding for a full evaluation. If further results are promising the program will be considered for the Australian Nurturing Families PCAP in Australia.”

In all, the paper offers valuable insights on the potential feasibility of the PCAP program in an Australian context. I wish the authors well in finalizing their paper towards publication.

Thanks!

Reviewer #2: The authors present a very well written paper on a well thought through and interesting study. However, the very small sample size is very concerning for a quantitative study even as the authors refer to it as a pilot study. My recommendation is that the authors provide a sample size calculation and the editor, in consultation with a statistician, gives this limitation serious consideration.

We would like to also thank reviewer #2 for their kind words and helpful suggestions!

Given the large difference between groups, if a replicate study was carried out a sample size calculation using a 99% confidence level, and a power of 90%, and means of .36 (before) and .77 after, using the larger sd of the after values (.11), the sample size needed would be two ([Sample Size Calculator For 2 Sample T Test - Benchmark Six Sigma](https://www.benchmarksixsigma.com/calculators/sample-size-calculator-for-2-sample-t-test/)). The very large difference in composite scale scores means a small sample size is adequate.

1. There is mention of Aborigines in the paper without an indication that this population is a focus of this study, which indeed from reading of the entire paper it is clear that this population is not the focus. I am concerned about this group being singled out as having a high FASD problem - perhaps the authors can also talk about other special populations where FASD prevalence is high rather than mention only one population.

You are correct, it is not a focus of this paper. Therefore, we have removed the reference to the FASD prevalence rate in the Fitzroy Valley in the Introduction (lines 66-68). We have added the number of people from other backgrounds in the description of the sample (Line 154), “Two clients identified as Australian, two as Aboriginal Australian, one Canadian, one Tongan and two were of unknown background.” We left in the reference to the additional training undertaken by the case-managers on line 255 as it is already within the context of other additional training they did including parenting and domestic violence.

2.a) The case-managers seem far too involved in the research process. They were both interventionists and researchers to a very small sample of women who were in need of resources that the case-managers were providing. All these dynamics create a huge potential for social desirability among the participants. The reason that the case-managers (rather than the researchers) conducted the baseline interviews is not currently not clear. Furthermore, the need for case managers to assist participants with recall, thus having to have them present in the interviews is also not convincing. In fact, the case-managers involvement seems to be viewed positively by the authors without reflection on how this may have biased the findings.

We have made it clearer in the Introduction that revealing information to a case-manager does not result in removal from the program at line 110, page 6, “Unlike many other AOD programs that withdraw help from clients who return to using AOD or have significant absences program, PCAP continues to provide support in these cases and case-managers “…never give up on a woman; women are never asked to leave the program because of relapse or setbacks.” p.3 [27].” Furthermore, it was part of our recruitment procedure to ensure that clients were aware that they did not have to take part in the research and non-participation would not affect their treatment at line 143 on page 7, “Due to the client vulnerability, it was emphasised that not taking part would not affect their position within the treatment program and they could withdraw at any time.”

Despite these efforts, we agree that social desirability can bias results and have added the following to the discussion at line 644, page 30, “Another potential issue arose from having case-managers involved with collecting baseline data and attending the follow-up interview. The ASI is an effective clinical instrument and we attempted to integrate it into normal clinical practice for baseline measurements. Efforts were made to reduce the impact of social desirability on client responses by make it clear during consent that their treatment would not be affected in any way by participation in the research study, or non-participation. It is also a key pillar of PCAP that clients are not refused treatment for revealing AOD, crime, or other personal details with case-managers adopting a non-judgemental attitude. To some extent, we believe that the trust between the case managers and the participants increased the likelihood of disclosure, thereby increasing the reliability of the findings. Despite this, the effects of social desirability may have biased client responses. In the future adopting a new approach from the US where supervised and ASI trained psychology Masters students conduct all interviews may be more appropriate.”

b) "WHFS approached the authors to evaluate a pilot PCAP program in Perth that had received funding for one case-manager." This text suggests that only one case manager was involved in the study but subsequent parts of the paper mention case-managers (plural) being involved. Some clarification here would be useful.

We have added at line 131 (page 7) that additional funding for a second case-manager was received during the pilot. “WHFS approached the authors to evaluate a pilot PCAP program in Perth that had initially received funding for one case-manager and during the pilot received additional funding for a second case-manager.” Also added at line 258 on page 12 that their role was, “These additional case-manager occasionally replaced the primary when they were unavailable.” At line 314 we have provided the average monthly hours delivered by the different case managers per client.

3. The description of the case manager interviews is too little - both in the methods and the findings. In some sections of the methods there is no mention of this component at all. At the moment it does not come across as an equally valuable part of this study and it is not well integrated with the rest of the paper. I think the paper presents too much at once, perhaps this component could be written up in a different paper. Relatedly, the design of the study does not currently meet the definition of a mixed methods study - i.e. as a third methodological approach alongside quantitative and qualitative methods. The authors would do well to reconsider use of this term for their study.

On the advice of both reviewers we have removed this section and other references to this aspect and made a note in the discussion that these results will be reported in an implementation paper at a later stage (Line 616, p. 29), “Further examination of these contextual issues, case-manager perceptions of the program and the full suite of implementation details will be undertaken after the first cohort of clients finish the program by applying the Consolidated Framework for Implementation Research (CFIR).”

We have removed any references to mixed methods.

Erng, M. N., Smirnov, A., & Reid, N. (2020). Prevention of Alcohol-Exposed Pregnancies and Fetal Alcohol Spectrum Disorder Among Pregnant and Postpartum Women: A Systematic Review. *Alcoholism: Clinical and Experimental Research, 44*(12), 2431-2448. doi:<https://doi.org/10.1111/acer.14489>
